# Moral Dilemmas in Hospitals: Which Shooting Victim Should Be Saved?

**DOI:** 10.3389/fpsyg.2022.770020

**Published:** 2022-03-25

**Authors:** Douglas J. Navarick, Kristen M. Moreno

**Affiliations:** Department of Psychology, California State University, Fullerton, Fullerton, CA, United States

**Keywords:** deservingness, responsibility, kinship bias, age bias, gender bias

## Abstract

Moral judgments can occur either in settings that call for impartiality or in settings that allow for partiality. How effective are impartiality settings such as hospitals in suppressing personal biases? Portrayed as decision-makers in an emergency department, 431 college students made judgments on which of two victims of a mass shooting should receive immediate, life-saving care. Patients differed in ways that could reveal biases, e.g., age (8 *vs.* 80 years), kinship (stranger *vs.* cousin), gender (female *vs.* male), and villain/hero (shooter *vs.* policeman who stopped him). Participants rated each patient’s moral deservingness to receive immediate care and the likelihood they would choose the patient. Both scales showed young favored over old, cousin (or daughter) over stranger, and policeman over shooter (largest difference). In a hospital-room scenario with high risk of injury from falling, age bias disappeared. With moderate fall risk, age bias reversed and kinship deservingness bias disappeared. Bias decreases when there is a decrease in severity of potential harm to the preferred stakeholder. Settings that call for impartiality are not reliable “boundary conditions” against expressions of bias. In the absence of explicit guidelines for allocating scarce resources, a systematic, objective method of random selection offers a potentially useful strategy.

## Introduction

Moral dilemmas are situations that require a choice between conflicting moral values or obligations. The COVID-19 pandemic has highlighted a particularly difficult dilemma that hospital emergency department personnel have faced in which they must decide which of several patients who are struggling to breathe will receive a ventilator or access to oxygen.

The allocation of scarce resources in emergency departments is a long-standing problem for which several standardized triage systems have been developed. The Emergency Severity Index (Gilboy et al., 2011) defines five levels of priority for care, with Level 1 (“Resuscitation”) the most urgent because without immediate treatment, death would be imminent, for example, due to “massive bleeding.”

Nurses and physicians may have no explicit guidelines to follow when two or more patients appear at the same time at the same level of severity and, in such situations, decisions would likely be vulnerable to a host of personal biases. The present study investigated the potential influence of a variety of biases in the context of a fictional, but realistic, scenario in which an emergency department is overwhelmed by an influx of victims of a mass shooting, many of whom are at Level 1. Participants take the role of the person who must decide which of two such patients will receive immediate care when resources are available to treat only one of them immediately. The patients differ in a way that would potentially reveal one of several types of bias when participants rate their reactions on two separate scales, one measuring how “morally deserving” a patient is to receive immediate care and the other measuring how strongly inclined the participant is to choose each patient.

### Partiality *vs*. Impartiality as Contexts for Moral Judgment

A hospital setting calls for impartiality in the way services are rendered, and there is an empirical basis for expecting that participants’ personal preferences when making triage decisions would be minimal, especially when decisions are expressed as a moral judgment. McManus et al. (2020, Study 5) presented scenarios in which either a stranger or a relative could be helped. There were two contexts for making the choice, one that allowed partiality and one that required impartiality because it went with the agent’s occupational role. The researchers’ Figure 5 shows results for the partiality context (e.g., seeing two people move into different apartments down the hall, one person a stranger the other a distant relative, and choosing to help one of them). The top row shows results with “Moral Goodness” as the dependent variable (as judged by a third party, not by the agent). In the Choice condition, ratings of moral goodness for helping kin were higher than for helping the stranger. Figure 6 shows results for the impartiality context (e.g., two undergraduate students ask a professor for an inconvenient, off-campus appointment to discuss graduate schools; one student is a stranger and the other is a distant relative). In the Choice condition, the pattern of ratings was the reverse of that in the partiality context, with higher ratings of goodness for helping the stranger than for helping kin. These results led the researchers to characterize contexts that require impartiality as “boundary conditions” (p. 1.) for the expression of personal bias.

A limitation of the experiment by McManus et al. (2020) is that it examined decision-making in a situation that had only minimally negative consequences for the stakeholder who was not chosen, for example, the relative would have to make a less convenient arrangement to meet with the professor. In a life-or-death situation, participants may well have judged choosing the distant relative to survive as being more morally good than choosing a stranger to survive. In that case the boundary condition for bias would be a context calling for impartiality combined with a relatively low, potential price to pay for being fair to the stakeholders.

Supporting this possibility is a study by Burnstein et al. (1994) who showed that, in a context allowing for partiality, a distinction between “everyday” situations and “life-or-death” situations (p. 776) is a key factor in predicting whether participants will be more inclined to help kin or a stranger. In the life-or-death situation participants were asked to imagine having to make a choice to rescue one of three people who were located in different rooms of a burning building. The characters differed in genetic proximity to the participant (labeled according to family relationships), age, and sex. Participants ranked the characters from the one they most likely would choose to the one they least likely would choose. In the everyday situation the same characters asked the participant to do them a small favor, such as pick up an item from a store, with time available for the participant to help only one of them.

As illustrated in the researchers’ Figure 2, in the life-or death situation the inclination to help dropped sharply as the degree of genetic relatedness to the participant decreased from 0.50 to 0.00, but in the everyday situation the decline was much less, with only a negligible difference (approximately 0.20 on a 3-point scale) between the tendencies to help at relatedness values of 0.125 (e.g., a cousin) and 0.00 (an acquaintance). The strength of this kinship effect under life-or-death conditions implies that a context of impartiality may not be sufficient to override it in a setting such as the Emergency Department of the present study, where in one condition the participant decides whether to give life-saving care either to their cousin or to a stranger.

**FIGURE 1:**
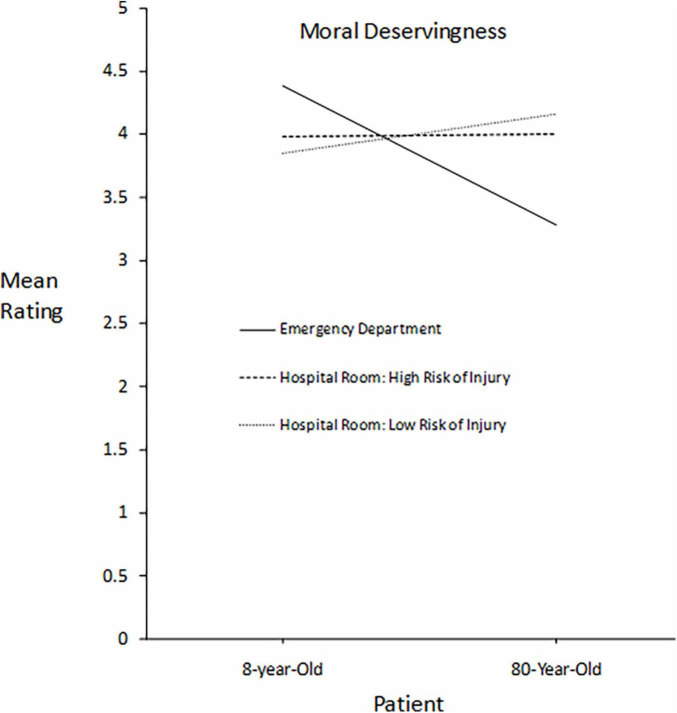
For patients differing in age, participants’ mean ratings of each patient’s moral deservingness of immediate care in three settings differing in severity of risk to the patients: death, high risk of injury, and lower risk of injury.

**FIGURE 2:**
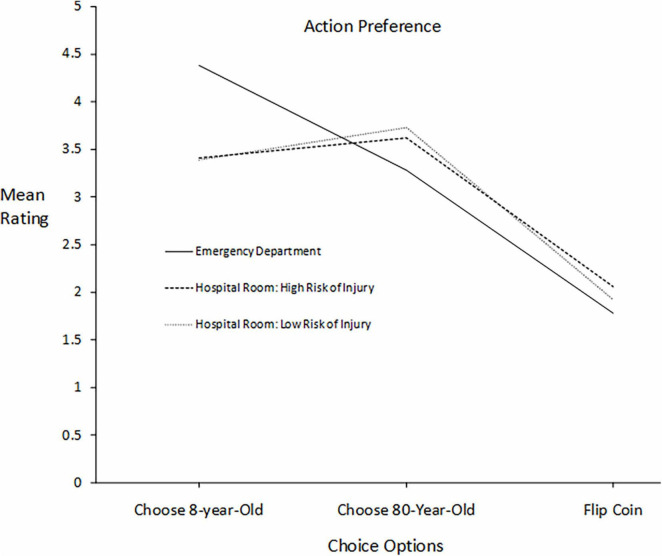
For patients differing in age, participants’ mean ratings of three courses of action in three settings differing in severity of risk to the patients: death, high risk of injury, and lower risk of injury.

### Varying the Level of Potential Risk to Patients

In a hospital setting there are obviously intermediate degrees of risk to a patient’s well-being between imminent death and inconvenience, a common risk being injury from a fall. To examine potential effects. of a decrease in severity of harm to a patient who does not receive immediate care, two pairs of patients were selected from the Emergency Department scenario for further analysis in a hospital room scenario involving fall risk: an 8 year-old girl *vs.* an 80-year- old woman, and the participant’s teenage cousin *vs.* a teenage stranger. As is common practice in hospitals the patients wear bracelets signifying their level of risk from a fall that could cause an injury. Two patients call at the same time for assistance getting out of bed to go to the bathroom but only one nurse is available to help them. The patient who is denied immediate assistance is likely to go unassisted. Participants rated two versions of this scenario, one where the patients’ bracelet signified high fall risk and one where it signified moderate fall risk.

**FIGURE 3:**
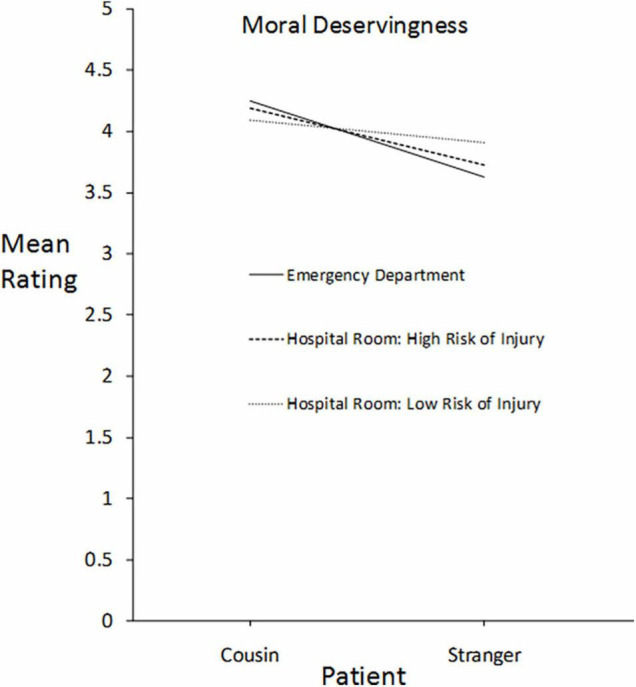
For patients differing in kinship relationship to the participants (cousin *vs.* stranger), participants’ mean ratings of each patient’s moral deservingness of immediate care in three settings differing in severity of risk to the patients: death, high risk of injury, and lower risk of injury.

The findings by Burnstein et al. (1994) suggest that there could be an attenuation of bias favoring the cousin as the level of risk to the unchosen patient decreased from life-or-death, to high fall risk, and to moderate fall risk. Additional findings from that study concerning age bias (illustrated in the researchers’ Figure 3) suggest an even stronger effect. In the life-or-death situation, there was a linear decrease in the inclination to help a person from infancy to 75 years of age, but in the everyday situation, there was a curvilinear relation, with the tendency to help highest at infancy and at 75 years, Directly related to the ages used in the present study is the comparison between 10 years and 75 years, which shows a reversal in bias from the younger to the older stakeholder when the threat to life is removed.

#### Broadening the Domain of Inclusive Fitness

An issue that needs to be considered when generalizing from Burnstein et al.’s (1994) findings to the present study is that their data are averages over conditions in which most of the stakeholders were portrayed as relatives of the participants, whereas here the patients differing in age were portrayed as strangers. Burnstein et al. (1994) interpreted their findings in terms of Hamilton’s (1964) *inclusive fitness* model of altruism, which holds that the tendency to help others depends on the extent to which the altruist shares genes with the beneficiary. If there is no information or cue that indicates a kinship relationship to the beneficiary, then without further assumptions the model would not clearly apply to strangers, and by extrapolation, the rationale for generalizing from relatives to strangers would be weakened.

However, it is important to recognize that almost all of one’s genes are identical to those of everyone else. According to the National Human Genome Research Institute (2018) “All human beings are 99.9 percent identical in their genetic makeup.” (NIH, 2021). Although the genetic relatedness of siblings is represented as 50%, this value implicitly refers only to the 0.10% of genes that vary across individuals. Altruistic tendencies are said to occur if they serve to preserve genes within this narrow range. It is an empirical question as to whether the same principle of gene preservation applies to the 99.90% of genes that everyone shares.

Supporting evidence would take the form of similarities in moral judgments and helping tendencies directed toward strangers and kin. To the extent that the present findings based on characters portrayed as strangers resembled those of Burnstein et al. (1994) based mostly on characters portrayed as relatives, the case for broadening the domain of inclusive fitness to universally shared genes would become more plausible. Such an account would also have the theoretical advantage of parsimony in that it would represent all genes as having a common tendency to replicate themselves through social interactions.

Alternative mechanisms are described in Trivers’ (1971) model of “reciprocal altruism” in which the altruist is said to benefit indirectly from helping non-kin without any advantage to a group with which the altruist identifies. “No concept of group advantage is necessary to explain the function of human altruistic behavior.” (Trivers, 1971, p. 48) However, it would be useful to consider the possibility that a group advantage could be present in the form of protecting humanity’s genes as a species. The wide variety of reciprocity mechanisms described by Trivers (1971) could have a common source in gene-preservation.

#### Distinguishing Between Moral Action and Moral Judgment

Another consideration when applying findings on bias from the study by Burnstein et al. (1994) is that they measured participants’ tendency to help a particular stakeholder rather than their judgment on how morally deserving that person would be to receive the help. Dispositions toward moral action and moral judgment can differ markedly. To act on a judgment requires having the social skills, resources, and fortitude to deal with the conflicts that one may encounter (Rest, 1986; Narvaez and Rest, 1995). Such conflicts could be internal as well as external. Tassy et al. (2013) presented participants with scenarios of 10 sacrificial moral dilemmas in which killing one person would save the lives of several others. There were separate questions related to moral judgment (“Is it acceptable to…in order to…”) and to moral action (“Would you…in order to…) (Tassy et al., 2013, p. 1). Participants were also assessed using a scale designed to measure traits characteristic of psychopathy. Higher scores on these traits, especially a trait of reduced affect, predicted higher numbers of participants endorsing sacrificial action but the scores did not correlate with judgments of acceptability.

As related to kinship bias, Kurzban et al. (2012) found that participants rated themselves more likely to sacrifice the life of one brother to save their five other brothers than to sacrifice one stranger to save five other strangers, but they rated these actions as equally morally wrong. Analogously, in the Emergency Department scenario, participants could rate a stranger and their cousin as being equally morally deserving of immediate care but rate themselves more likely to choose their cousin.

### Background on Potential Sources of Bias

The pairs of characters and the types of bias they were intended to measure were as follows: **(1)** *age bias*: an 8-year-old girl *vs.* an 80-year-old woman; **(2)** *kinship bias*: your teenage daughter *vs.* a teenage girl you don’t know; **(3)** *kinship bias with fewer shared genes*: a teenage girl who is your cousin *vs.* a teenage girl you don’t know; **(4)** *gender bias*: a teenage boy you don’t know *vs.* a teenage girl you don’t know; **(5)** *bias against villains and favoring heroes (“just deserts”)*: the shooter responsible for mass causalities *vs.* the policeman who shot and stopped him; **(6)** *in-group/out-group bias*: a man who lives on the streets *vs.* a man who has a home. Presented below is a selection of studies related to each type of bias.

#### Age Bias

Under life-or-death conditions, the linear increase in participants’ tendency to help others as their age decreased (Burnstein et al., 1994) appears to parallel a more general, widely held set of dispositions. Axt et al. (2014) used Implicit Association Tests to examine social evaluation hierarchies in over 200,000 participants. Based on the patterns of associations, the researchers inferred social hierarchies for “races,” religions, and age. Although hierarchies differed somewhat in terms of the most highly valued group (one’s own “race” or religion), participants in all demographic categories showed the most positive associations for children, followed by young adults, middle-age adults, and then older adults.

The relatively low evaluation of the elderly is reflected in sacrificial dilemmas. Kawai et al. (2014) varied the age of the target to be sacrificed in two versions of the frequently studied “trolley” dilemma in which a runaway trolley threatens to kill several people down the tracks if not stopped or diverted. In the “Footbridge” version a person would have to be thrown off the bridge onto the tracks to derail the trolley and in the “Switch” version the trolley could be diverted to another track by operating a switch, which would then kill the target without personal contact. The former version typically produces a stronger emotional reaction and is judged to be less morally acceptable. The targets varied in age and physical condition: a 70-year-old man, a 20-year-old man, a 20-year-old disabled man, and a 5-year-old boy. In both versions, participants were found to give higher ratings of appropriateness for sacrificing the elderly man than the other targets, who did not differ significantly from one another.

#### Kinship Bias

Sacrificial dilemmas describe life-or-death situations and, based on Burnstein et al.’s (1994) demonstration of the importance of this factor, such situations would be directly relevant to choosing which patient in an emergency department should receive immediate, life-saving care, as the patient not chosen would essentially be sacrificed to save the other patient.

In an experiment by Navarick (2021), participants were presented with several such dilemmas where five beneficiaries of sacrificial action were described as the participants’ children, cousins or strangers. The participants’ rated both how morally right and how morally wrong it would be to sacrifice the target—a firefighter hero or a notorious bank robber—to save each group. Going from strangers to cousins to children, ratings of right increased and ratings of wrong decreased with each target. Sensitivity to changes in the beneficiaries’ identities was stronger for ratings of right than ratings of wrong, possibly because the term “morally right” induced greater attention to the prospect of saving lives.

These effects of kinship relationship on moral judgment complement Burnstein et al.’s (1994) results on the tendency to help, with the former directly relevant to ratings of moral deservingness and the latter to the scale of inclination to choose each patient. However, for both studies, the context was one that allowed for partiality. The hospital setting calls for impartiality and could prevent expressions of kinship bias.

#### Gender Bias

**FIGURE 4:**
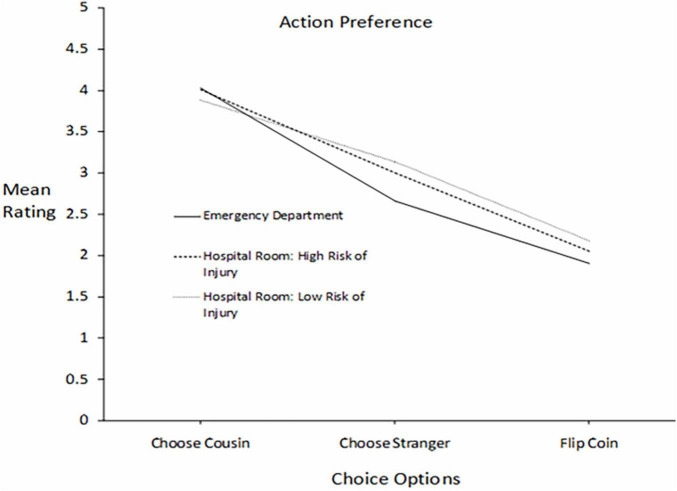
For patients differing in kinship relationship to the participants (cousin *vs.* stranger), mean ratings of three courses of action in three settings differing in severity of risk to the patients: death, high risk of injury, and lower risk of injury.

When it comes to protecting people from harm, a common cultural bias is to give women higher priority than men, a predisposition known as “moral chivalry” (FeldmanHall et al., 2016). In a Footbridge version of the Trolley dilemma, when participants were given a choice between pushing a male or a female bystander off the bridge to save the people further down the tracks, participants were seven times more likely to choose the male than the female target (FeldmanHall et al., 2016, Figure 1). In Burnstein et al.’s (1994) model of altruism based on inclusive fitness, the tendency to help females is predicted to be higher both in life-or-death and everyday situations but for different reasons. In the everyday situation, the rationale is essentially moral chivalry whereas in the life-or-death situation the rationale is women’s greater average reproductive potential up to the age of menopause, at which point females would no longer be favored over males. These predictions were supported, as illustrated in the researchers’ Figures 4, 5.

There is also evidence to support either the absence of a bias or a bias that favors males over females. An extensive study by Weissbourd et al. (2015) employed a variety of methods—primarily surveys, interviews, and focus groups—to examine attitudes of middle and high school students and adults toward gender, especially as related to leadership roles. In one survey conducted with over 19,000 students, when a bias was present, both female and male teens favored males over females as political leaders, but the majority of both genders expressed no preference (females: 69%, males 56%). The data suggest that if a bias was found in the present study, it would tend to favor males. With regard to the absence of a bias in most participants, it is noteworthy that the differences found by Burnstein et al. (1994) were relatively small, with maximum differences ranging from approximately 0.2 to 0.3 on a 3-point scale.

#### In-Group/Out-Group Bias

Using fictional sacrificial dilemmas, Cikara et al. (2010) found that participants judged it more acceptable to sacrifice one person to save five people if the person being sacrificed was perceived as an out-group member than as an in-group member. An extreme out-group member tended to be someone who was perceived as being neither warm nor competent and was epitomized by the homeless. Participants judged it most acceptable to sacrifice the homeless and least acceptable to save them. Although these social evaluations could vary markedly across research samples, the study by Cikara et al. (2010) provides a basis for representing out-group and in-group members in terms of being either homeless or having a home. In contrast, based on their application of inclusive fitness theory, Burnstein et al. (1994) expected a stronger tendency to help poor kin than wealthy kin in an everyday situation (because it would be seen as more moral or appropriate) but in a life-or-death situation, the pattern would be the reverse for distant kin and there would be no bias involving close kin. These predictions were supported, as illustrated in the researchers’ Figure 9.

#### Hero vs. Villain and the Modeling of Just Deserts

In research on how users of narrative entertainment react to the characters that are portrayed, the Affective Disposition Theory (Zillmann and Cantor, 1972) states that the initial reaction is to form a positive or negative impression of the characters based on judgments of their moral character. Although other possible sources of these impressions have been suggested, it is generally assumed that an audience has a strong preference for a story that shows a positive outcome for the “good guy” or hero and a negative outcome for the villain (Grizzard et al., 2020). On this basis, one may expect participants in the present study to rate the perpetrator of a mass shooting as less deserving of immediate care than the policeman who stopped him.

However, a more precise prediction that also has implications for the other characters can be derived from a model of deservingness judgments proposed by Feather (1999), which will be described in detail under section “Discussion.” The premise of the model is causal attribution, the perception of a person as having direct responsibility for creating a situation, which then becomes the basis for judging the person’s deservingness of the consequences: “I have assumed that a person cannot be judged to deserve an outcome for which he or she is not responsible” (p. 92). Only the shooter/policeman pair met this requirement. Personal characteristics of an individual (e.g., kinship relationship, likability, being perceived as a member of one’s in-group) would act as moderator variables, influencing the judge’s assessment of the level of positivity or negativity of the action or outcome. In the absence of a perception of personal responsibility, the model would not apply directly to such characteristics and a judgment of moral deservingness would then depend on other kinds of beliefs, such as a fundamental human right to life shared equally by all.

### Hypotheses and Rationale

Hypotheses are stated in terms of participants’ mean ratings on separate scales representing their judgments of moral deservingness and their inclination to choose a particular course of action.

Each patient was rated separately, as illustrated by the following sample of scales taken verbatim from the survey. The examples refer to one of the patients and the other one is indicated in brackets.

**The patients are an 8-year-old girl *vs.* an 80-year-old woman.**

On a scale from 0 to 5, please enter a number in the box that represents how MORALLY DESERVING you feel the 8-year-old girl [80-year-old woman] is to be treated first (0 = not at all deserving, 5 = extremely deserving):

**The following questions ask about what you feel you would probably do. There are three options: choose the 8-year-old girl, choose the 80-year-old woman, or “just flip a coin” (choose randomly).**

On a scale from 0 to 5, please enter a number in the box that represents how likely you are to choose the 8-year-old girl [80-year-old woman, “just flip a coin”] (0 = definitely would not, 5 = definitely would):

#### Emergency Department: Judgments of Moral Deservingness

*Hypothesis 1:* For five of the six pairs, measures of moral deservingness for immediate care will be equal because the patients had no causal responsibility for being injured and they would likely be seen as sharing a basic human right to life. For one pair—the shooter *vs.* the policeman who shot and stopped him—ratings of moral deservingness will be much higher for the policeman than for the perpetrator based both on Feather’s (1999) model and the generalization from fictional narratives that audiences typically prefer to see positive consequences for heroes and negative consequences for villains.

#### Emergency Department: Action Preferences

*Hypothesis 2:* The previously discussed biases will be shown for most pairs of patients. Participants will give a higher mean rating of their inclination to choose: (a) the 8-year-old girl than the 80-year-old woman, (b) their teenage daughter than a teenage girl they do not know, (c) a teenage girl who is their cousin than a teenage girl they do not know, (d) the policeman who shot and stopped the shooter who caused mass casualties than the shooter, (e) a man who has a home than a man who lives on the streets.

*Hypothesis 3:* For the teenage boy and the teenage girl whom the participants do not know, there will be no clear difference on either scale based on the conflicting findings and small effects that were discussed in the summaries of previous studies.

#### Hospital Room: Risk of Injury From a Fall Without Immediate Attention

Two pairs of patients were selected from the Emergency Department scenario for further analysis when there was a lower severity of risk to the patients if immediate care was not provided: 8- *vs.* 80-year old and cousin vs. stranger.

The patients wear bracelets signifying their level of risk from a fall that could cause an injury. Two patients call at the same time for assistance getting out of bed to go to the bathroom but only one nurse is available to help them. The patient who is denied immediate assistance is likely to go unassisted. Participants rated two versions of this scenario, one where the patients’ bracelet signified high fall risk and one where it signified a moderate all risk. These analyses were exploratory in nature but there seemed to be a sufficient basis for stating a hypothesis regarding the option to avoid choosing a patient and to resolve the issue by flipping a coin.

*Hypothesis 4:* As the severity of potential harm to the unchosen patient decreases from death, to high risk of injury, to moderate risk of injury, participants’ ratings of their likelihood of choosing the coin option will increase. Flipping the coin would represent a form of escape from an “avoidance-avoidance conflict.” Participants would tend to avoid directly exposing their preferred patient to harm by denying them assistance but also tend to avoid being biased in a setting that calls for impartiality. As the severity of harm to the preferred patient decreases and becomes more tolerable, the coin option becomes more attractive as a way to avoid both being biased and being directly responsible for harming the preferred patient.

## Materials and Methods

The SPSS data file for this study is available using the following link: https://osf.io/8gnyk/?view_only=617b0797b28444e09184dffd271bab74.

The verbatim survey text is provided in the Supplementary Files.

### Participants

A total of 431 undergraduates at California State University, Fullerton participated in the survey during the Fall (2019) and Spring (2020) semesters. Participants chose the study from a list presented on the Psychology Department’s online research management system. The title was “Moral Dilemmas in Hospitals.” Forty participants accessed the survey an additional 49 times, and these duplicate cases were deleted from the data analysis. The majority of the students (73.6%) came from introductory psychology classes and participated to fulfill a research hours requirement; all but 1 of the remaining students came from other lower-division courses and upper-division psychology courses.

### Demographics

Most participants (75.8%) were female, and 24.0% were male, with 1 participant identifying as “Other.” Participants had an average age of 20.35 years (*SD* = 3.84, range 18–61). Researchers presented the following question to characterize the ethnic backgrounds of the participants: “What ethnicity or ethnicities do you identify as?”. Participants were able to choose from the following ethnic examples. However, they were not limited to them and responses using other labels were categorized as closely as possible to the ones presented for summary purposes: African American/African 2.2%, American 13.4%, Anglo-Saxon 0.2%, Asian 12.1%, Asian-American 9.7%, Caucasian 3.7%, European 4.0%, Hispanic 48.3%, Middle Eastern 3.7%, Native American 0.5%, Pacific Islander 0.7%, Other (specific countries: Russia, Mexico, Italy, Vietnam) 1.5%. Additionally, 6.3% of the 431 participants did not respond with an ethnicity label and were excluded from the distribution of ethnicities.

### Experimental Design and Approach to Data Analysis

There were two main independent variables: Patient Pairs (six pairs, each representing a specific form of bias as previously discussed) and Severity of Risk (three levels of potential harm to the patient who was not chosen for immediate care): high (death), moderate (high risk of falling and being injured), and low (moderate risk of falling and being injured). The high-risk level was studied in the context of the Emergency Department scenario and the moderate- and low-risk levels were studied in the context of the hospital room scenarios. There were two dependent variables: ratings of each patient’s moral deservingness to receive immediate care, and ratings of the participant’s likelihood of choosing each patient or flipping a coin instead.

There were two types of data analysis. One type focused on the Emergency Department and used *t*-tests to assess the significance of ratings differences within pairs of patients. Some *t*-tests were also conducted to see if one pair had a significantly larger difference than another pair.

To examine the severity of risk variable, three pairs were selected for additional presentation in the hospital room scenarios: 8 *vs*. 80 years of age, cousin *vs.* stranger, and homeless man *vs.* man with a home. Only the first two pairs showed significant biases in the Emergency Department and only they were analyzed further in the two hospital room scenarios. For each pair there were two types of repeated measures ANOVAs, each of which had two independent variables. One type of ANOVA had Setting and Patient (each patient of the pair) as independent variables. The other type of ANOVA had Setting as one variable and Choice Options (choosing each of the two patients and flipping a coin) as the second variable.

### Procedure

The verbatim survey, including the scenarios and all questions, are provided in the Supplementary Files. The survey was administered online through SurveyMonkey. The first page presented the Informed Consent Statement. The following pages consisted of questions about pairs of patients that were segmented into 3 blocks of pages: Block 1 (pages 2 through 8), Block 2 (pages 9 through 12), Block 3 (pages 13 through 16).

The first block asked questions regarding the Emergency Department scenario. Page 2 described the scenario and the participants’ general task of expressing their views on scales from 0 to 5:

“You are working in the Emergency Room at the local hospital. Emergency rooms have “triage” rules for incoming patients that assess the severity of each patient’s condition based on medical indicators and assign that person a priority number for treatment.”

“In each of the following cases, two patients arrive at the same time with multiple gunshot wounds due to a mass shooting. They are equally severe cases and both are designated as “Level 1,” which means that they are both in immediate danger of dying from their wounds and should be treated next. However, due to the many patients who have arrived at the Emergency Room, resources are available to treat just one of them immediately, and it is likely that the patient who is not treated immediately will die.”

“Your hospital has provided personnel with no guidelines for what to do when two or more patients receive the same priority number. In each of the following cases, the two patients are briefly described and you are asked to express your views on a series of rating scales that range from 0 to 5. “

Subsequent pages incorporated questions that measured the six forms of bias. On each of these pages (from 3 to 8) one pair of characters was presented along with rating scales for moral deservingness and personal choice of action. The second block, consisting of pages 9 through 12, introduced the Hospital Room Priorities scenario with the “highest level of danger” from falling:

“You are a nurse working at your local hospital. The hospital has a set of guidelines for patients who are considered ‘Fall Risk.’ Each patient is required to wear a hospital band that has a color representing their level of danger from falling. It is mandatory that you use the band colors to prioritize the amount of attention you give to patients when they are out of bed.”

“Two patients call to you at the same time for immediate assistance going to the bathroom. Both patients have on yellow bands indicating the highest level of danger. A nurse must be present any time they are out of bed. They say they can’t wait. Due to a shortage of staff, you are the only nurse who is currently available to accompany patients to the bathroom. If you don’t help them, they may go to the bathroom alone and fall.”

“In each of the following cases, the patients are briefly described, and you are asked to express your views on a series of rating scales that range from 0 to 5.”

Pages 10 through 12, presented the three pairs of characters. Each page presented one pair of characters and both rating scales. The third block (pages 13 through 16) had questions on the Hospital Room scenario with the lower risk of falling. Page 13 explained the scenario and the general task:

“Suppose that the patients are wearing an orange band, indicating that they have a moderate risk of falling. They have been instructed to call for help if they feel they need it when they get out of bed. Unlike patients with the yellow band, they do not require continuous attention when they are in the bathroom or in a wheelchair. In each of the following cases, the patients are briefly described, and you are asked to express your views on a series of rating scales that range from 0 to 5.” Each of the next three pages presented questions on one pair of patients and the two rating scales.

Pages within the blocks that had questions about the character pairs were presented in different random orders across participants. When the order of pages in a block was randomized, the order of presentation of the character pairs within that severity level was randomized.

To indicate a rating, the participants were asked to type a number from 0 to 5 in a textbox that was labeled according to a specific patient, and in the case of the question of choice of action, there was an additional option to “just flip a coin.” For the questions on moral deservingness, the order of these textboxes (i.e., patients in a pair) changed randomly. For the choice of action questions, the order of the patients and the coin option changed randomly. Examples of the questions were presented under “Hypotheses and Rationale” in the section “Introduction.”

#### Personality Scales, Questions on Demographics, and Debriefing Statement

Subsequent pages consisted of several personality scales, the data from which were not analyzed here but were collected for possible future use, either by the present researchers or by others, to identify correlates of individual differences in the participants’ ratings. The scales are: the “Moral Identity Scale” (Aquino and Reed, 2002), the “Oxford Utilitarianism Scale” (Kahane et al., 2017), and the “Centrality of Religiosity Scale” (Huber and Huber, 2012). The questions are included in the copy of the survey text in the Appendix and the data are included in the SPSS file that can be accessed using the link presented earlier.

After the personality scales came a demographics page that asked participants for their age, gender, psychology class, and one or more self-created labels that indicated ethnic identity. The next page presented the debriefing statement and final page thanked participants for taking the survey.

## Results

The analysis of bias in Emergency Department ratings consisted of 19 comparisons, all of which were evaluated using paired *t*-tests. Multiple statistical tests involving the same dependent variable are vulnerable to Type 1 errors (rejection of a true null hypothesis). A conservative correction was applied in the form of a Bonferroni correction (initial alpha level/number of comparisons) combining comparisons from both the deservingness and the choice of action scales:0.05/19 = 0.0026. Values of *p* equal to or less than.002 were considered the strongest evidence for statistical significance. Sample sizes changed slightly across comparisons due to some participants’ occasionally omitting required ratings. Resources were sufficient to obtain samples of approximately *N* = 425, which allowed detection of an effect size as small as *dz* = 0.14 at a 0.80 level of power (a level typically recommended for use in psychology), with an alpha level = 0.05.

Values of *d* were obtained using the calculator for repeated measures designs at *https://www.psychometrica.de/effect_size.html*. Values reported here are those referred to in the calculator as *d_*Repeated Measures, pooled*_*, which uses “the pooled standard deviation, controlling for the intercorrelation of both groups.”

### Personal Responsibility and Demographics as Factors in Deservingness Ratings

Hypothesis 1 was only partially supported. Paired-sample *t*-tests were conducted for each of the 6 pairs of characters. Just two rather than all five of the character pairs that differed only in demographic characteristics failed to show a significant difference: gender bias, where the mean rating for “teenage boy you don’t know” (*M* = 3.85, *SD* = 1.35, *N* = 424) did not differ significantly from the mean for a “teenage girl you don’t know” *(M* = 4.05, *SD* = 2.41, *N* = 424), [*t(*423*)* = 1.927, *p* = 0.055, *d* = 0.09], and in-group/out-group bias related to homelessness, where the mean rating for “man who lives on the street” (*M* = 3.78, *SD* = 1.35, *N* = 423) did not differ significantly from the mean for “a man who has a home” (*M* = 3.84, *SD* = 1.30, *N* = 423), [*t(*422*)* = –0.988, *p* = 0.324, *d* = 0.05]. In contrast, the policeman who stopped the violence was judged to be significantly and far more deserving of immediate care (*M* = 4.48, *SD* = 1.02, *N* = 425) than the shooter who created the crisis (*M* = 1.90, *SD* = 2.23, *N* = 425), *t*(424) = –20.543, *p* < 0.001, *d* = 0.65, demonstrating a bias for “just deserts.”

The remaining character pairs demonstrated biases related to the age of the patients and to their kinship relationship to the decision-maker (i.e., the participant): (1) ratings for the 8-year-old (*M* = 4.36, *SD* = 1.05, *N* = 428) significantly exceeded ratings for the 80-year-old (*M* = 3.28, *SD* = 1.48, *N* = 428), *t*(427) = 14.287, *p* < 0.001, *d* = 0.57. demonstrating an age bias; (2) ratings for “your teenage daughter” (*M* = 4.35, *SD* = 1.19, *N* = 426) significantly exceeded ratings for a “teenage girl you don’t know” (*M* = 3.55, *SD* = 1.48, *N* = 426), *t*(425) = 11.352, *p* < 0.001, *d* = 0.51, demonstrating a kinship bias involving a close relationship; and (3) ratings for “your teenage cousin” (*M* = 4.25, *SD* = 1.82, *N* = 426) significantly exceeded ratings for a “teenage girl you don’t know” (*M* = 3.63, *SD* = 2.89, *N* = 426), *t*(425) = 4.047, *p* < 0.001, *d* = 0.15, demonstrating a kinship bias involving a more distant relationship.

#### Size of Ratings Differences: Comparison Across Character Pairs

It is clear from the data that the size of the difference between ratings for the policeman/shooter pair exceeded that for the other pairs. To assess the significance of the size of these ratings differences, the following procedures were used. A new variable was created for each pair of characters representing the difference between the ratings given by each participant. Then, using paired *t-*tests, the mean difference score for the policeman/shooter pair was compared to the mean difference score for each of these three pairs of characters. In all cases, the policeman/shooter pair had a significantly higher difference score, indicating that personal responsibility was a stronger factor in ratings of moral deservingness for immediate care than age or kinship relationship. For the comparison with the 8/80-year-old pair, (*M* = 1.50, *SD* = 2.75), *t*(422) = 11.205, *p* < 0.001, *d* = 0.42; for the comparison with daughter/teen stranger, (*M* = 1.78, *SD* = 2.81), *t*(422) = 13.025, *p* < 0.001, *d* = 0.46; for the comparison with teenage cousin/teen stranger, (*M* = 1.97, *SD* = 3.93), *t*(422) = 10.304, *p* < 0.001, *d* = 0.37.

### Personal Responsibility and Demographics as Factors in Ratings of Personal Choice of Action

Hypothesis 2 stated that ratings of personal choice of action would be sensitive to bias in five of the six-character pairs, the exception being the teen boy/teen girl pair. Hypothesis 3 stated that that there would be no significant difference for this pair. Evidence for bias was found in all of the character pairs except the one measuring in-group/out-group bias, where the mean rating for “man who lives on street” (*M* = 3.29, *SD* = 2.02, *N* = 423) did not differ significantly from the mean for “man who has a home” (*M* = 3.41, *SD* = 1.34, *N* = 423*), t(*422*)* = –1.117, *p* = 0.265, *d* = 0.04.

The other five character pairs demonstrated the following biases (favored character italicized): (1) ratings for the *8-year-old (M* = 4.16, *SD* = 1.15, *N* = 427*)* significantly exceeded ratings for the 80-year-old (*M* = 2.52, *SD* = 1.41, *N* = 427), *t(*426*)* = 18.333, *p* < *0.001*, *d* = 0.62, demonstrating an age bias; (2) ratings for “*your teenage daughter*” (*M* = 4.48, *SD* = 2.74, *N* = 426) significantly exceeded ratings for a “teenage girl you don’t know” (*M* = 2.50, *SD* = 1.53, *N* = 426), *t*(425) = 12.948, *p* < 0.001, *d* = 0.44, demonstrating a kinship bias involving a close relationship; (3) ratings for “*your teenage cousin*” (*M* = 4.03, *SD* = 1.26, *N* = 426) significantly exceeded ratings for a “teenage girl you don’t know” (*M* = 2.66, *SD* = 1.38, *N* = 426), *t(*425*)* = 16.030, *p* < 0.001, *d* = 0.46, demonstrating a kinship bias involving a more distant relationship; (4) contrary to Hypothesis 3, a “*teenage girl you don’t know*” (*M* = 3.38, *SD* = 1.44, *N* = 424) significantly exceeded ratings for a “teenage boy you don’t know” (*M* = 3.21, *SD* = 1.44, *N* = 424), *t(*423*)* = 3.077, *p* = 0.002, *d* = 0.19, implying gender bias, which contrasted with the absence of evidence for bias in the moral deservingness ratings (further analyzed, below), and (5) ratings for *policeman who stopped the violence* (*M* = 4.25, *SD* = 1.20, *N* = 424) significantly exceeded ratings for the shooter responsible for mass casualties (*M* = 1.59, *SD* = 1.47, *N* = 424), *t(*423*)* = –25.187, *p* < 0.001, *d* = 0.76, demonstrating a bias for “just deserts.”

Regarding the comparison between teen boy and teen girl, further analysis showed that the significantly higher mean for teen girl on the choice scale, and no significant difference on the deservingness scale, did not represent the choice pattern of most participants. Each participant could show one of three possible outcomes when their ratings were compared for boy and girl (boy – girl): a higher rating for boy than girl (+), a higher rating for girl than boy (–), and equal ratings (0), a total of nine possible combinations across the two scales. The frequency of each combination was calculated and by far the most frequent combination (approximately 68% of the sample) had equal ratings on the two scales, which is consistent with Hypothesis 3. Only about 14% of participants had a pattern showing no difference on the deservingness scale and a higher rating for the girl on the choice scale.

#### Size of Ratings Differences: Comparison Across Character Pairs

The difference score for the policeman/shooter pair significantly exceeded the difference scores for the four other character pairs that showed a significant bias. Listed below are the results of the *t*-tests showing the mean difference between the difference scores, the *SD* for the mean difference score, and the *t*-test values.

For the comparisons with the 8/80-year-old pair *M* = 1.04, *SD* = 2.44, *t*(420) = 8.719, *p* < 0.001, *d* = 0.35; for the comparison with daughter/teen stranger, *M* = 0.66, *SD* = 3.27, *t*(421) = 4.162, *p* < 0.001, *d* = 0.17; for the comparison with teenage cousin/teen stranger, *M* = 1.29, *SD* = 2.33, *t*(421) = 11.343, *p* < 0.001, *d* = 0.47; for the comparison with teenage boy/teenage girl, *M* = 2.81, *SD* = 2.50, *t*(420) = 23.064, *p* < 0.001, *d* = 0.68.

### Comparisons Between Emergency Department (Risk to Patients: Death) and Hospital Room (Risk to Patients: Injury From Fall)

Two pairs of characters were used to investigate possible changes in bias going from the Emergency Department to the hospital room: an 8-year-old girl and an 80-year-old woman, and a teenage girl who is your cousin and teenage girl you don’t know. As no bias was demonstrated in the Emergency Department between a man who lives on the streets and a man who has a home, this pair was not included in the analysis of changes in bias (but it was included in the analysis of changes in ratings of the flip-a-coin option, discussed below).

#### The Choice Not to Choose: “Just Flip a Coin”

Hypothesis 4 stated that as the severity of risk to the unchosen patient decreased from death to high risk of injury to moderate risk of injury, there would be an increase in participants’ ratings of the chances that they would flip a coin rather than choose a patient. The hypothesis was partially supported for the two pairs that exhibited significant initial bias in the Emergency Department—8/80 and cousin/stranger—but not the pair that exhibited no initial bias—homeless man/man with home.

To reduce the statistical risk of a Type 1 error, a Bonferroni correction was applied based on the 6 comparisons in this section, resulting in an alpha level of 0.05/6 = 0.008. Significance was considered achieved when the probability found was 0.008 or less.

For the 8/80 pair, the mean in the Emergency Department (*M* = 1.78, *SD* = 1.80, *N* = 417) was significantly lower than the mean in the hospital room when there was a high risk of falling (*M* = 2.06, *SD* = 1.86, *N* = 417), *t*(416) = 3.100, *p* = 0.002, *d* = 0.16, but there was no significant difference when there was a moderate risk of falling—emergency department (*M* = 1.76, *SD* = 1.79, *N* = 411), hospital room (*M* = 1.92, *SD* = 1.82, *N* = 411), *t*(410) = 1.704, *p* = 0.089, *d* = 0.08.

For teen cousin/teen stranger, the mean in the Emergency Department (*M* = 1.90, *SD* = 1.83, *N* = 418) did not differ significantly (based on the Bonferroni modified alpha of 0.008) from the mean in the hospital room when there was a high risk of falling— (*M* = 2.16, *SD* = 2.44, *N* = 418), *t*(417) = 2.32, *p* = 0.021, *d* = 0.11—but it did differ significantly when there was a moderate risk of falling— Emergency Department (*M* = 1.89, *SD* = 1.84, *N* = 412) *vs.* hospital room (*M* = 2.18, *SD* = 1.98, *N* = 412), *t*(411) = 3.150, *p* = 0.002, *d* = 0.15.

For homeless man/man with home, the mean in the Emergency Department (*M* = 2.59, *SD* = 1.91, *N* = 416) had no significant difference from a high risk of falling—hospital room *(M* = 2.69, *SD* = 1.99, *N* = 416*), t(*415*)* = 1.168, *p* = 0.243, *d* = 0.07. Additionally, the mean in the emergency department (*M* = 2.58, *SD* = 1.90, *N* = 412) did not differ significantly from the mean with a moderate risk of falling (*M* = 2.67, *SD* = 1.99, *N* = 412), *t*(411) = 1.045, *p* = 0.297, *d* = 0.05. It is noteworthy that this pair, which showed no bias in the Emergency Department, also had a higher initial rating of the coin option than the other pairs and had subsequent ratings that held steady at the initial level.

#### Age Bias: Effects of Severity of Risk on Ratings of Moral Deservingness and Action Preference

Figure 1 presents participants’ mean ratings of moral deservingness for immediate care for the 8- and 80-year-old patients. The solid line represents the Emergency Department, the dashed line represents the Hospital Room condition with a high fall risk, and the dotted line represents the Hospital Room condition with a moderate fall risk. The central finding illustrated by the graph is that the bias favoring the 8-year-old in the Emergency Department was not present in the Hospital Room conditions. Furthermore, a paired *t* test comparing the 8-year-old and the 80-year-old in the condition with moderate fall risk found that the mean for the 8-year-old (*M* = 3.85, *SD* = 1.32, *N* = 414) was significantly *lower* than the mean for the 80-year-old (*M* = 4.16, *SD* = 2.82, *N* = 414), *t*(413) = 2.261, *p* = 0.024, *d* = 0.10, indicating that the age bias favoring the 8-year-old in the Emergency Department was reversed when the risk to the patients’ welfare was sufficiently reduced.

A repeated measures ANOVA was performed, and the effects were assessed as a multivariate test based on the chart provided by SPSS. Pillai’s trace was used as a correction to *F* values for potential departures from assumptions, such as homogeneity of variance.

There was a significant main effect of Patient, *F*(1,410) = 14.337, *p* < 0.001, indicating that the 8-year-old was favored over the 80-year-old when ratings were averaged over the three settings. There was also a significant main effect Setting, *F*(2,409) = 12.108, *p* < 0.001, indicating that ratings of moral deservingness were somewhat lower in the Emergency Department when the ratings were averaged over the two patients, reflecting the low ratings given to the 80-year-old in the Emergency Department. Most importantly, there was a significant Patient*Setting interaction, *F*(2,409) = 70.105, *p* < 0.001, indicating that the effect of Patient depended on the Setting of the scenario, as shown in Figure 1.

Figure 2 presents participants’ mean ratings of their inclination to choose a particular course of action: choose the 8-year-old, choose the 80-year-old, and flip a coin. The format of the graph is the same as that for Figure 1. Focusing on just the two patients, the pattern is identical to that for moral deservingness ratings.

A repeated measures ANOVA was conducted and assessed in the same manner as it was for ratings of moral deservingness. There was a significant main effect of Choice Options, *F*(2,406) = 158.491, *p* < 0.001 averaged across Setting, and a significant main effect of Setting averaged across Choice Options, *F*(2,406) = 15.169, *p* < 0.001. The effect most relatable to moral deservingness ratings is the interaction between Choice Options and Setting. which was significant, *F*(4,404) = 66.717, *p* < 0.001 Interpretation of the effect is complicated by the inclusion of the third option, flip a coin, but it is attributable mostly to the elimination of the preference for the 8-year-old in the Hospital Room conditions. Variation in the slopes from 8-year-old to 80-year-old was much greater than the variation in slopes from the 80-year-old to the coin option. As with the deservingness ratings, a paired *t-*test for the condition with moderate fall risk found that the mean for the 8-year-old (*M* = 3.39, *SD* = 2.45, *N* = 414) was significantly *lower* than the mean for the 80-year-old (*M* = 3.73, *SD* = 1.39, *N* = 414), *t*(413) = 2.47, *p* = 0.014, *d* = 0.09, indicating that the age bias favoring the 8-year-old for help in the life-or-death situation was reversed when the risk to the patients’ welfare was sufficiently reduced.

### Kinship Bias: Ratings of Moral Deservingness and Personal Choice of Action

Figure 3 presents participants’ mean ratings of moral deservingness for immediate care for their teenage cousin and a teenage stranger in the Emergency Department (solid line), in the hospital room with a high fall risk (dashed line), and in the hospital room with a low fall risk (dotted line).

A repeated measures ANOVA was performed, and the effects were assessed as multivariate tests using the Pillai’s trace correction. There was a significant main effect of Patient, *F*(1,411) = 28.901, *p* < 0.001, indicating that teenage cousin was favored over the teenage stranger when ratings were averaged over the three settings. There was no significant main effect of Setting, *F*(2,410) = 0.419, *p* = 0.658, indicating that, when ratings of moral deservingness were averaged over patients, the ratings did not vary significantly across the three settings. However, the setting did have an effect on ratings in that a significant interaction occurred between Patient and Setting, *F*(2,410) = 3.038, *p* = 0.049. The difference between patients in the Emergency Department was eliminated in the hospital room with a moderate fall risk (lowest level of potential harm). A paired *t* test comparing Cousin and Stranger in the moderate fall-risk condition found that the mean for teenage cousin (*M* = 4.09, *SD* = 1.22, *N* = 413) did not differ significantly from the mean for teenage stranger (*M* = 3.91, *SD* = 2.77, *N* = 413). *t*(412) = 1.416, *p* = 0.157, *d* = 0.06, a trend resembling that for age bias but not to the point of reversing the bias favoring the cousin. The plausibility of this apparent null effect is supported by the significant interaction between Patient and Setting.

Figure 4 presents participants’ mean ratings of their inclination to choose a particular course of action: choose teenage cousin, choose teenage stranger, and flip a coin. The format of the graph is the same as that for Figure 3.

A repeated measures ANOVA was conducted and was assessed in the same manner as it was for ratings in moral deservingness. There was a significant main effect of Choice Options, *F*(2,409) = 182.434, *p* < 0.001 averaged across Setting, and a significant main effect of Setting averaged across Choice Options, *F*(2,409) = 19.030, *p* < 0.001. As with the ratings of moral deservingness, the interaction between Choice Option and Setting was significant, *F*(4,407) = 11.605, *p* < 0.001, suggesting a slight decrease in bias (i.e., a decrease in slopes) going from the Emergency Department to the hospital room conditions. However, in contrast to the deservingness ratings, the kinship bias favoring the cousin for help in the life-or-death situation was maintained when the threat was reduced to a moderate risk of injury: (*M* = 3.88, *SD* = 1.27, *N* = 414) was significantly higher than the mean for the stranger (*M* = 3.13, *SD* = 1.30, *N* = 413), *t*(412) = 9,765, *p* < 0.001, *d* = 0.39.

## Discussion

Overall, the data support the generalization that, at least for first-person judgments, a context of impartiality is not sufficient to eliminate expressions of bias (cf. McManus et al., 2020). For example, in the Emergency Department, the risk to the relative was death, and a cousin was strongly favored over a stranger. The cousin was also favored in the hospital room condition with a high fall risk. However, the bias disappeared in ratings of moral deservingness in the hospital room condition with a moderate fall risk.

A stronger effect occurred with age bias, influencing measures of both moral deservingness and the inclination to choose a patient. The 8-year-old was favored over the 80-year-old in the Emergency Department but the bias disappeared in the hospital room with a high risk of injury from a fall, and the bias significantly reversed in favor of the 80-year-old when there was a moderate fall risk. This reversal replicates and extends the pattern found by Burnstein et al. (1994; summarized under Introduction) comparing the tendencies to help a 10-year-old *vs.* a 75-year-old in a life-or-death rescue attempt as compared to an everyday favor. The reversal effect generalized from a partiality context involving mostly kin to an impartiality context involving strangers at a much higher level of risk to the well-being of the stakeholders when the reversal occurred. The price of fairness appears to be the decisive factor in whether personal biases influence decisions in a context that requires impartiality.

That these effects observed with kin also occurred with strangers supports a broader application of Burnstein et al.’s (1994) inclusive fitness model of altruism to include strangers. The premise of the model is that altruism reflects a tendency to behave in ways that increase the chances of passing along one’s genes to future generations. Considering that 99.9% of one’s genes are identical to those of everyone else, it is reasonable to suppose that the same tendency would occur even in the absence of information or cues indicating that the beneficiaries would be kin. In effect, all human beings are kin, one family.

### Assessment of Hypotheses

For the Emergency Department, Hypotheses 1 and 3 stated that ratings of moral deservingness for immediate care would be equal within all pairs of patients except the shooter/policeman pair because only they performed actions that caused them to be in the hospital and in need of immediate care. The policeman was expected to receive higher ratings of deservingness than the shooter because these consequences would be seen as their “just deserts.” This aspect of the hypothesis was supported, along with the findings of no significant differences for teen girl/teen boy and homeless man/man with a home. The underlying theory can be represented within a formal model of deservingness judgments proposed by Feather (1999), which will be discussed under the heading, “Modeling Just Deserts.”

However, several other pairs of characters showed a significant difference within the pair—the 8-year-old was favored over the 80-year-old (age bias), and the teen cousin and teen daughter were each favored over teen strangers (kinship bias). It is noteworthy that the shooter/policeman pair had a significantly greater difference in ratings of deservingness than any of these other pairs, showing that causal responsibility for being in the Emergency Department was a more important factor in deservingness ratings than the demographic characteristics of the patients.

Hypothesis 2 stated the expectation that personal biases would show up in behavioral tendencies, the participants’ ratings of their inclination to choose a particular patient. This hypothesis was supported in that the action ratings paralleled the deservingness ratings for the shooter/policeman pair and the pairs that showed an age or kinship bias.

Hypothesis 4 stated that as risk decreased going from the Emergency Department to the hospital room settings involving high and moderate risks of injury from a fall, there would be an increase in the inclination to avoid a choice between patients and choose an option to “just flip a coin.” In this way, the decision-maker could escape from an avoidance-avoidance conflict of being responsible for harm to the favored patient and being biased in a setting calling for impartiality. Consistent with the hypothesis, there was a small but significant increase in ratings favoring the coin option across these three conditions.

Hospitals and other organizations that seek to minimize the influence of personal bias in the allocation of scarce resources may find it useful to consider an analogous process that is systematic (e.g., computer-generated random numbers) and objective (at least two employees would monitor each step of the selection process). The data suggest that the choice not to choose tends to become more acceptable to decision-makers when the risks to the well-being of stakeholders are not a matter of life-or-death. However, even under such conditions, it would be useful to consider why it would be preferable for an employee to make a personal decision if there was no objective basis for distinguishing between the stakeholders.

#### Modeling “Just Deserts”

In describing his model of the “The Structure of Deservingness,” Feather (1999, pp. 92–93), emphasized the point that the model applies only when a person is seen as performing an action that has resulted in an outcome that is to be judged as deserved or not deserved. The outcome in the present study would be receiving immediate care in the Emergency Department (or in one of the hospital room conditions). Personal characteristics of an individual (e.g., kinship relationship, likability, being perceived as a member of one’s in-group) would act as moderator variables that alter the evaluation of the action or outcome.

The Structural Model consists of four elements:

1. PERSON—the one who judges deservingness.
2. OTHER—the one who is being judged.
3. ACTION—that resulted in the outcome at issue
4. OUTCOME—that was caused by the other’s action and is being assessed.

In the present study, the four elements would be as follows:

1. PERSON = participant
2. OTHER = patient
3. ACTION = none except for shooter and policeman
4. OUTCOME = immediate care in hospital

The model has two rules for assessing the deservingness of an outcome:

1. An outcome is judged as *deserved* when both the ACTION and OUTCOME are viewed as positive or both are viewed as negative.
2. An outcome is judged as *undeserved* when the ACTION is viewed as positive and the OUTCOME as negative, or the ACTION is viewed as negative and the OUTCOME as positive.

The policeman was known to have performed a protective action *(positive*) that would have a deserved outcome if he received immediate care (*positive*). The shooter was known to have performed a destructive action (*negative*) that would have an undeserved outcome if he received immediate care (*positive*). The high rating of deservingness for the policeman and the low rating of deservingness for the shooter support this model.

A related but less comprehensive model is the “Path” model of blame proposed by Monroe and Malle (2017, 2019). As in Feather’s (1999) model, an initial assessment is made of the agent’s causal responsibility for an event, and if there is no perceived responsibility, there will be no judgment of blame. If processing continues, the next step is a determination of intentionality. If the event is perceived to have been caused intentionally, then the agent’s reasons are considered, and if the event is perceived to have been caused unintentionally, then factors are considered related to preventability. The focus on blame allows only for assessment of negative consequences for the agent, whereas the deservingness model also allows for positive consequences. An integration of the two models could represent processes underlying judgments of both blame and praise.

### Limitations and Future Directions

The presence and degree of some biases may vary across samples drawn from regions with different histories and values. As indicated in the Demographics section under Method, the sample was highly diverse in terms of ethnicities, and the study was conducted at a public university that explicitly values and promotes diversity and tolerance. These influences could have been reflected in the lack of clear biases related to the character pairs that differed in sex and in wealth status (homeless *vs*. having a home). Replications involving a variety of cultural contexts would be useful. It would also be useful to include both 1st- and 3rd-party judgments. The present study presents the perspective of participants who imagine being the one who decides which patient will be chosen for immediate care. There were strong biases favoring kin (cousin, child) over a stranger. For such pairs, 3rd-party judgments seem likely to differ, especially for judgments of moral deservingness. For age bias, 3rd-party and 1st-party judgments seem likely to agree because they do not specifically favor the decision-maker.

## Conclusion

The present study points to a variety of personal biases that could influence decisions when hospital personnel are called upon to allocate scarce resources to patients and there is no clear, objective basis for making a choice. In such cases, hospital ethics committees may find it useful to consider the strategy discussed earlier of requiring the use of a systematic, objective process of random selection.

## Data Availability Statement

The datasets presented in this study can be found in online repositories. The names of the repository/repositories and accession number(s) can be found in the article/Supplementary Material.

## Ethics Statement

The studies involving human participants were reviewed and approved by Institutional Review Board California State University, Fullerton. The patients/participants provided their written informed consent to participate in this study.

## Author Contributions

Both authors conceptualized and designed the experiment. DN had primary responsibility for preparing the manuscript. KM had primary responsibility for collecting the data and conducting the statistical analyses.

## Conflict of Interest

The authors declare that the research was conducted in the absence of any commercial or financial relationships that could be construed as a potential conflict of interest.

## Publisher’s Note

All claims expressed in this article are solely those of the authors and do not necessarily represent those of their affiliated organizations, or those of the publisher, the editors and the reviewers. Any product that may be evaluated in this article, or claim that may be made by its manufacturer, is not guaranteed or endorsed by the publisher.
