# Supplementary material for: Moral Dilemmas in Hospitals: Which Shooting Victim Should Be Saved?
Source: Front Psychol. 2022 Mar 25;13:770020. doi: 10.3389/fpsyg.2022.770020 (PMC8989733; doi:10.3389/fpsyg.2022.770020)
Supplement: Supplementary file 1 [file Data_Sheet_1.docx]

**Appendix: Verbatim Survey Text**

P1: INFORMED CONSENT STATEMENT

You must be 18 years of age or older and a CSUF undergraduate student to participate in this

study.

You will first be reading several descriptions of situations in which you are portrayed as

someone who needs to make a decision. For each situation you will be asked several questions about how you view your options. You will then be given several questionnaires that ask about various personal characteristics, including religious attitudes. Your participation is voluntary and if you want to discontinue your participation, you are free to do so without a penalty; you will still receive your credit for 0.75 hour of participation.

This protocol contains no foreseeable risks. Confidentiality of your research records will be maintained to the extent provided by the law. Your answers on the survey are protected by our use of anonymous survey forms that cannot be linked to you. We collect identifying information about you at the beginning of the session to give you credit for your participation, but this information can in no way be linked to your responses on the survey. These anonymous records are kept indefinitely on the personal and/or office computers of the researchers (as well as the web server that administers the survey). We hold onto them so that we or other researchers can analyze the information for additional studies. Your name will not appear in any published or final product.

There are no physical risks associated with your participation in this research. However, due to the violent content of some of the scenarios, you may feel uncomfortable while reading or thinking about them or answering the questions. Please note that you can skip any scenarios that make you uncomfortable.

Although you will receive no direct benefits, this research may help us better understand how people make moral decisions that affect other people. The purposes of the research will be presented after you have completed your participation.

.......

Please click NEXT to start the survey.

P2: Emergency Room Priorities

You are working in the Emergency Room at the local hospital. Emergency rooms have “triage” rules for incoming patients that assess the severity of each patient’s condition based on medical indicators and assign that person a priority number for treatment.

In each of the following cases, two patients arrive at the same time with multiple gunshot

wounds due to a mass shooting. They are equally severe cases and both are designated as “Level 1”, which means that they are both in immediate danger of dying from their wounds and should be treated next. However, due to the many patients who have arrived at the Emergency Room, resources are available to treat just one of them immediately, and it is likely that the patient who is not treated immediately will die.

Your hospital has provided personnel with no guidelines for what to do when two or more patients receive the same priority number. In each of the following cases, the two patients are briefly described and you are asked to express your views on a series of rating scales that range from 0to 5.

P3: Emergency Room Priorities

1. The patients are an 8-year-old girl vs. an 80-year-old woman.

On a scale from 0 to 5, please enter a number in the box that represents how MORALLY

DESERVING you feel the 8-year-old girl is to be treated first (0 = not at all deserving, 5 = extremely deserving):

On a scale from 0 to 5, please enter a number in the box that represents how MORALLY

DESERVING you feel the 80-year-old woman is to be treated first (0 = not at all deserving, 5 = extremely deserving):

2. The following questions ask about what you feel you would probably do. There are 3

options: choose the 8-year-old girl, choose the 80-year-old woman, or “just flip a coin”

(choose randomly).

On a scale from 0 to 5, please enter a number in the box that represents how likely you are to choose the 8-year-old girl (0 = definitely would not, 5 = definitely would):

On a scale from 0 to 5, please enter a number in the box that represents how likely you are to choose the 80-year-old woman (0 = definitely would not, 5 = definitely would):

On a scale from 0 to 5, please enter a number in the box that represents how likely you are to “just flip a coin” (0 = definitely would not, 5 = definitely would):

P4: Emergency Room Priorities

3. The patients are your teenage daughter vs. a teenage girl you don’t know.

On a scale from 0 to 5, please enter a number in the box that represents how MORALLY

DESERVING you feel your teenage daughter is to be treated first (0 = not at all deserving, 5 = extremely deserving):

On a scale from 0 to 5, please enter a number in the box that represents how MORALLY

DESERVING you feel the teenage girl you don’t know is to be treated first (0 = not at all

deserving, 5 = extremely deserving):

4. The following questions ask about what you feel you would probably do. There are 3

options: choose your teenage daughter, choose the teenage girl you don’t know, or “just flip a coin” (choose randomly).

On a scale from 0 to 5, please enter a number in the box that represents how likely you are to choose your teenage daughter (0 = definitely would not, 5 = definitely would):

On a scale from 0 to 5, please enter a number in the box that represents how likely you are to choose the teenage girl you don’t know (0 = definitely would not, 5 = definitely would):

On a scale from 0 to 5, please enter a number in the box that represents how likely you are to “just flip a coin” (0 = definitely would not, 5 = definitely would):

P5: Emergency Room Priorities

5. The patients are a teenage girl who is your cousin vs. a teenage girl you don’t know.

On a scale from 0 to 5, please enter a number in the box that represents how MORALLY

DESERVING you feel your teenage cousin is to be treated first (0 = not at all deserving, 5 = extremely deserving):

On a scale from 0 to 5, please enter a number in the box that represents how MORALLY

DESERVING you feel the teenage girl you don’t know is to be treated first (0 = not at all

deserving, 5 = extremely deserving):

6. The following questions ask about what you feel you would probably do. There are 3

options: choose your teenage cousin, choose the teenage girl you don’t know, or “just flip a coin” (choose randomly).

On a scale from 0 to 5, please enter a number in the box that represents how likely you are to choose your teenage cousin (0 = definitely would not, 5 = definitely would):

On a scale from 0 to 5, please enter a number in the box that represents how likely you are to choose the teenage girl you don’t know (0 = definitely would not, 5 = definitely would):

On a scale from 0 to 5, please enter a number in the box that represents how likely you are to “just flip a coin” (0 = definitely would not, 5 = definitely would):

P6: Emergency Room Priorities

7. The patients are a teenage boy you don’t know vs. a teenage girl you don’t know.

On a scale from 0 to 5, please enter a number in the box that represents how MORALLY

DESERVING you feel the teenage boy you don’t know is to be treated first (0 = not at all

deserving, 5 = extremely deserving):

On a scale from 0 to 5, please enter a number in the box that represents how MORALLY

DESERVING you feel the teenage girl you don’t know is to be treated first (0 = not at all

deserving, 5 = extremely deserving):

8. The following questions ask about what you feel you would probably do. There are 3

options: choose the teenage boy you don’t know, choose the teenage girl you don’t know, or “just flip a coin” (choose randomly).

On a scale from 0 to 5, please enter a number in the box that represents how likely you are to choose the teenage boy you don’t know (0 = definitely would not, 5 = definitely would):

On a scale from 0 to 5, please enter a number in the box that represents how likely you are to choose the teenage girl you don’t know (0 = definitely would not, 5 = definitely would):

On a scale from 0 to 5, please enter a number in the box that represents how likely you are to “just flip a coin” (0 = definitely would not, 5 = definitely would):

P7: Emergency Room Priorities

9. The patients are the shooter vs. a policeman who shot and stopped him.

On a scale from 0 to 5, please enter a number in the box that represents how MORALLY

DESERVING you feel the shooter is to be treated first (0 = not at all deserving, 5 = extremely deserving):

On a scale from 0 to 5, please enter a number in the box that represents how MORALLY

DESERVING you feel the policeman is to be treated first (0 = not at all deserving, 5 = extremely deserving):

10. The following questions ask about what you feel you would probably do. There are 3

options: choose the shooter, choose the policeman who shot and stopped him, or “just flip a coin” (choose randomly).

On a scale from 0 to 5, please enter a number in the box that represents how likely you are to choose the shooter (0 = definitely would not, 5 = definitely would):

On a scale from 0 to 5, please enter a number in the box that represents how likely you are to choose the policeman (0 = definitely would not, 5 = definitely would):

On a scale from 0 to 5, please enter a number in the box that represents how likely you are to “just flip a coin” (0 = definitely would not, 5 = definitely would):

P8: Emergency Room Priorities

11. The patients are a man who lives on the streets vs. a man who has a home.

On a scale from 0 to 5, please enter a number in the box that represents how MORALLY

DESERVING you feel the man who lives on the streets is to be treated first (0 = not at all

deserving, 5 = extremely deserving):

On a scale from 0 to 5, please enter a number in the box that represents how MORALLY

DESERVING you feel the man who has a home is to be treated first (0 = not at all deserving, 5 = extremely deserving):

12. The following questions ask about what you feel you would probably do. There are 3

options: choose the man who lives on the streets, choose the man who has a home, or “just flip a coin” (choose randomly).

On a scale from 0 to 5, please enter a number in the box that represents how likely you are to choose the man who lives on the streets (0 = definitely would not, 5 = definitely would):

On a scale from 0 to 5, please enter a number in the box that represents how likely you are to choose the man who has a home (0 = definitely would not, 5 = definitely would):

On a scale from 0 to 5, please enter a number in the box that represents how likely you are to “just flip a coin” (0 = definitely would not, 5 = definitely would):

P9: Hospital Room Priorities

You are a nurse working at your local hospital. The hospital has a set of guidelines for patients who are considered “Fall Risk”. Each patient is required to wear a hospital band that has a color representing their level of danger from falling. It is mandatory that you use the band colors to prioritize the amount of attention you give to patients when they are out of bed.

Two patients call to you at the same time for immediate assistance going to the bathroom. Both patients have on yellow bands indicating the highest level of danger. A nurse must be present any time they are out of bed. They say they can’t wait. Due to a shortage of staff, you are the only nurse who is currently available to accompany patients to the bathroom. If you don’t help them, they may go to the bathroom alone and fall.

In each of the following cases, the patients are briefly described, and you are asked to express your views on a series of rating scales that range from 0 to 5.

P10: Hospital Room Priorities

13. The patients are an 8-year-old girl vs. an 80-year-old woman.

On a scale from 0 to 5, please enter a number in the box that represents how MORALLY

DESERVING you feel the 8-year-old girl is to be cared for first (0 = not at all deserving, 5 = extremely deserving):

On a scale from 0 to 5, please enter a number in the box that represents how MORALLY

DESERVING you feel the 80-year-old woman is to be cared for first (0 = not at all deserving, 5 = extremely deserving):

14. The following questions ask about what you feel you would probably do. There are 3

options: choose the 8-year-old girl, choose the 80-year-old woman, or “just flip a coin”

(choose randomly).

On a scale from 0 to 5, please enter a number in the box that represents how likely you are to choose the 8-year-old girl (0 = definitely would not, 5 = definitely would):

On a scale from 0 to 5, please enter a number in the box that represents how likely you are to choose the 80-year-old woman (0 = definitely would not, 5 = definitely would):

On a scale from 0 to 5, please enter a number in the box that represents how likely you are to “just flip a coin” (0 = definitely would not, 5 = definitely would):

P11: Hospital Room Priorities

15. The patients are a teenage girl who is your cousin vs. a teenage girl you don’t know.

On a scale from 0 to 5, please enter a number in the box that represents how MORALLY

DESERVING you feel the teenage girl who is your cousin is to be cared for first (0 = not at all deserving, 5 = extremely deserving):

On a scale from 0 to 5, please enter a number in the box that represents how MORALLY

DESERVING you feel the teenage girl you don’t know is to be cared for first (0 = not at all deserving, 5 = extremely deserving):

16. The following questions ask about what you feel you would probably do. There are 3

options: choose the teenage girl who is your cousin, choose the teenage girl you don’t know, or “just flip a coin” (choose randomly).

On a scale from 0 to 5, please enter a number in the box that represents how likely you are to choose the teenage girl who is your cousin (0 = definitely would not, 5 = definitely would):

On a scale from 0 to 5, please enter a number in the box that represents how likely you are to choose the teenage girl you don’t know (0 = definitely would not, 5 = definitely would):

On a scale from 0 to 5, please enter a number in the box that represents how likely you are to “just flip a coin” (0 = definitely would not, 5 = definitely would):

P12: Hospital Room Priorities

17. The patients are a man who lives on the streets vs. a man who has a home.

On a scale from 0 to 5, please enter a number in the box that represents how MORALLY

DESERVING you feel the man who lives on the streets is to be cared for first (0 = not at all deserving, 5 = extremely deserving):

On a scale from 0 to 5, please enter a number in the box that represents how MORALLY

DESERVING you feel the man who has a home is to be cared for first (0 = not at all deserving, 5 = extremely deserving):

18. The following questions ask about what you feel you would probably do. There are 3

options: choose the man who lives on the streets, choose the man who has a home, or “just flip a coin” (choose randomly).

On a scale from 0 to 5, please enter a number in the box that represents how likely you are to choose the man who lives on the streets (0 = definitely would not, 5 = definitely would):

On a scale from 0 to 5, please enter a number in the box that represents how likely you are to choose the man who has a home (0 = definitely would not, 5 = definitely would):

On a scale from 0 to 5, please enter a number in the box that represents how likely you are to “just flip a coin” (0 = definitely would not, 5 = definitely would):

P13: Hospital Room Priorities

Suppose that the patients are wearing an orange band, indicating that they have a moderate risk of falling. They have been instructed to call for help if they feel they need it when they get out of bed. Unlike patients with the yellow band, they do not require continuous attention when they are in the bathroom or in a wheelchair. In each of the following cases, the patients are briefly described, and you are asked to express your views on a series of rating scales that range from 0 to 5.

P14: Hospital Room Priorities

19. The patients are an 8-year-old girl vs. an 80-year-old woman.

On a scale from 0 to 5, please enter a number in the box that represents how MORALLY

DESERVING you feel the 8-year-old girl is to be cared for first (0 = not at all deserving, 5 = extremely deserving):

On a scale from 0 to 5, please enter a number in the box that represents how MORALLY

DESERVING you feel the 80-year-old woman is to be cared for first (0 = not at all deserving, 5 = extremely deserving):

20. The following questions ask about what you feel you would probably do. There are 3

options: choose the 8-year-old girl, choose the 80-year-old woman, or “just flip a coin”

(choose randomly).

On a scale from 0 to 5, please enter a number in the box that represents how likely you are to choose the 8-year-old girl (0 = definitely would not, 5 = definitely would):

On a scale from 0 to 5, please enter a number in the box that represents how likely you are to choose the 80-year-old woman (0 = definitely would not, 5 = definitely would):

On a scale from 0 to 5, please enter a number in the box that represents how likely you are to “just flip a coin” (0 = definitely would not, 5 = definitely would):

P15: Hospital Room Priorities

21. The patients are a teenage girl who is your cousin vs. a teenage girl you don’t know.

On a scale from 0 to 5, please enter a number in the box that represents how MORALLY

DESERVING you feel the teenage girl who is your cousin is to be cared for first (0 = not at all deserving, 5 = extremely deserving):

On a scale from 0 to 5, please enter a number in the box that represents how MORALLY

DESERVING you feel the teenage girl you don’t know is to be cared for first (0 = not at all deserving, 5 = extremely deserving):

22. The following questions ask about what you feel you would probably do. There are 3

options: choose the teenage girl who is your cousin, choose the teenage girl you don’t know, or “just flip a coin” (choose randomly).

On a scale from 0 to 5, please enter a number in the box that represents how likely you are to choose the teenage girl who is your cousin (0 = definitely would not, 5 = definitely would):

On a scale from 0 to 5, please enter a number in the box that represents how likely you are to choose the teenage girl you don’t know (0 = definitely would not, 5 = definitely would):

On a scale from 0 to 5, please enter a number in the box that represents how likely you are to “just flip a coin” (0 = definitely would not, 5 = definitely would):

P16: Hospital Room Priorities

23. The patients are a man who lives on the streets vs. a man who has a home.

On a scale from 0 to 5, please enter a number in the box that represents how MORALLY

DESERVING you feel the man who lives on the streets is to be cared for first (0 = not at all deserving, 5 = extremely deserving):

On a scale from 0 to 5, please enter a number in the box that represents how MORALLY

DESERVING you feel the man who has a home is to be cared for first (0 = not at all deserving, 5 = extremely deserving):

24. The following questions ask about what you feel you would probably do. There are 3

options: choose the man who lives on the streets, choose the man who has a home, or “just flip a coin” (choose randomly).

On a scale from 0 to 5, please enter a number in the box that represents how likely you are to choose the man who lives on the streets (0 = definitely would not, 5 = definitely would):

On a scale from 0 to 5, please enter a number in the box that represents how likely you are to choose the man who has a home (0 = definitely would not, 5 = definitely would):

On a scale from 0 to 5, please enter a number in the box that represents how likely you are to “just flip a coin” (0 = definitely would not, 5 = definitely would):

P17: Your Self-Concept

Please feel free to skip any of these personal questions that you would prefer not to answer.

Listed below are some characteristics that might describe a person:

Caring, Compassionate, Fair, Friendly, Generous, Helpful, Hardworking, Honest, and

Kind

The person with these characteristics could be you or it could be someone else. For a moment, visualize in your mind the kind of person who has these characteristics. Imagine how that person would think, feel, and act. When you have a clear image of what this person would be like, answer the following questions from 1 (strongly disagree) to 5 (strongly agree).

Please feel free to skip any of these personal questions that you would prefer not to answer. gree).

25. It would make me feel good to be a person who has these characteristics.

1 Strongly Disagree

2 3 4

5 Strongly Agree

26. Being someone who has these characteristics is an important part of who I am.

1 Strongly Disagree

2 3 4

5 Strongly Agree

27. I often wear clothes that identify me as having these characteristics.

1 Strongly Disagree

2 3 4

5 Strongly Agree

28. I would be ashamed to be a person who had these characteristics.

1 Strongly Disagree

2 3 4

5 Strongly Agree

29. The types of things I do in my spare time (e.g., hobbies) clearly identify me as having

these characteristics.

1 Strongly Disagree

2 3 4

5 Strongly Agree

30. The kinds of books and magazines that I read identify me as having these

characteristics.

1 Strongly Disagree

2 3 4

5 Strongly Agree

32. The fact that I have these characteristics is communicated to others by my membership

in certain organizations.

1 Strongly Disagree

2 3 4

5 Strongly Agree

33. I am actively involved in activities that communicate to others that I have these

characteristics.

1 Strongly Disagree

2 3 4

5 Strongly Agree

34. I strongly desire to have these characteristics.

1 Strongly Disagree

2 3 4

5 Strongly Agree

P18: Your Moral Views

Please feel free to skip any of these personal questions that you would prefer not to answer.

Please indicate how much you agree or disagree with each of the following statements (1 =

strongly disagree, 4 = neither agree nor disagree, 7 = strongly agree)

35. If the only way to save another person’s life during an emergency is to sacrifice one’s

own leg, then one is morally required to make this sacrifice.

Strongly Disagree

1

2

3

Neither Agree nor Disagree

4

5

6

Strongly Agree

7

36. From a moral point of view, we should feel obliged to give one of our kidneys to a

person with kidney failure since we do not need two kidneys to survive, but really only one to be healthy.

Strongly Disagree

1

2

3

Neither Agree nor Disagree

4

5

6

Strongly Agree

7

37. From a moral perspective, people should care about the well-being of all human beings on the planet equally; they should not favor the well-being of people who are especially close to them either physically or emotionally.

Strongly Disagree

1

2

3

Neither Agree nor Disagree

4

5

6

Strongly Agree

7

38. It is just as wrong to fail to help someone as it is to actively harm them yourself.

Strongly Disagree

1

2

3

Neither Agree nor Disagree

4

5

6

Strongly Agree

7

39. It is morally wrong to keep money that one doesn’t really need if one can donate it to

causes that provide effective help to those who will benefit a great deal.

Strongly Disagree

1

2

3

Neither Agree nor Disagree

4

5

6

Strongly Agree

7

40. It is morally right to harm an innocent person if harming them is a necessary means to

helping several other innocent people.

Strongly Disagree

1

2

3

Neither Agree nor

Disagree

4

5

6

Strongly Agree

7

41. If the only way to ensure the overall well-being and happiness of the people is through the use of political oppression for a short, limited period, then political oppression should be used.

Strongly Disagree

1

2

3

Neither Agree nor Disagree

4

5

6

Strongly Agree

7

42. It is permissible to torture an innocent person if this would be necessary to provide

information to prevent a bomb going off that would kill hundreds of people.

Strongly Disagree

1

2

3

Neither Agree nor Disagree

4

5

6

Strongly Agree

7

43. Sometimes it is morally necessary for innocent people to die as collateral damage—if

more people are saved overall.

Strongly Disagree

1

2

3

Neither Agree nor Disagree

4

5

6

Strongly Agree

7

P19: Your Religious Beliefs and Practices

Please feel free to skip any of these personal questions that you would prefer not to answer.

The following questions inquire about your religious beliefs and practices. For each item,

indicate the most appropriate answer for you by checking the appropriate answer to the question.

READ EACH ITEM CAREFULLY BEFORE RESPONDING. Answer as honestly as you can.

Thank you.

44. How often do you think about religious issues?

Never

Rarely

Occasionally

Often

Very Often

45. To what extent do you believe that God or something divine exists?

Not At All

Not Very Much

Moderately

Quite A Bit

Very Much So

46. How often do you take part in religious services?

Never

Less often

A few times a year

One or three times a month

Once a week

More than once a week

47. How often do you pray?

Never

Less often

A few times a year

One or three times a month

Once a week

More than once a week

Once a day

Several times a day

48. How often do you experience situations in which you have the feeling that God or

something divine intervenes in your life?

Never

Rarely

Occasionally

Often

Very Often

49. How interested are you in learning more about religious topics?

Not At All

Not Very Much

Moderately

Quite A Bit

Very Much So

50. To what extend do you believe in an afterlife—e.g. immortality of the soul, resurrection

of the dead or reincarnation?

Not At All

Not Very Much

Moderately

Quite A Bit

Very Much So

51. How important is to take part in religious services?

Not At All

Not Very Much

Moderately

Quite A Bit

Very Much So

52. How important is personal prayer for you?

Not At All Not Very Much Moderately Quite A Bit Very Much So

Not At All

Not Very Much

Moderately

Quite A Bit

Very Much So

53. How often do you experience situations in which you have the feeling that God or

something divine wants to communicate or to reveal something to you?

Never

Rarely

Occasionally

Often

Very Often

54. How often do you keep yourself informed about religious questions through radio,

television, internet, newspapers, or books?

Never

Rarely

Occasionally

Often

Very Often

55. In your opinion, how probable is it that a higher power really exists?

Not At All

Not Very Much

Moderately

Quite A Bit

Very Much So

56. How important is it for you to be connected to a religious community?

Not At All

Not Very Much

Moderately

Quite A Bit

Very Much So

57. How often do you pray spontaneously when inspired by daily situations?

Never

Rarely

Occasionally

Often

Very Often

58. How often do you experience situations in which you have the feeling that God or

something divine is present?

Never

Rarely

Occasionally

Often

Very Often

P20: Gender, Age, Ethnicity

Please feel free to skip any of these personal questions that you would prefer not to answer.

59. Please indicate your gender:

FEMALE

MALE

OTHER

60. Please indicate your age (number of years):

61. Please indicate the class you are taking:

PSYC ...

PSYC ...

PSYC ...

PSYC ...

PSYC....

PSYC ...

PSYC ...

PSYC ...

PSYC ...

PSYC ...

OTHER PSYCHOLOGY COURSE

OTHER COURSE IN A DIFFERENT DEPARTMENT

62. What ethnicity or ethnicities do you identify as? Possible examples include, but are not limited to, the following options:

African

African American

American

Anglo Saxon

Asian

Asian American

European

Hispanic/Latino

Middle Eastern

Native American

Pacific Islander

frican

African American

American

Anglo Saxon

Asian

Asian American

European

Hispanic/Latino

Middle Eastern

Native American

Pacific Islander

Please type your answer or answers in the text boxes, below, in any order.

Ethnicity:

Ethnicity:

Ethnicity:

Ethnicity:

Ethnicity:

63. Please provide any additional information in the text box, below, that you feel describes

your ethnic identity.

P21: Explanation of Study

When caring for patients, nurses may have to make decisions that involve value judgments on their part due to an absence of guidelines from the hospital, or they may have clear rules but a lack of resources to implement them. In this study, we are asking undergraduates ...

to make moral judgments about moral judgments about what choices they feel should be made between two patients who need immediate care but resources are available to care for only one of them immediately.

Specifically, we asked how "morally deserving" each character in a pair was to receive

immediate treatment. We expect that these deservingness ratings will be very similar because it will be seen as a human right. However, when it comes to taking action, there are likely to be biases toward one or the other patient. We chose pairs of characters to study various kinds of biases:

1) The patients are an 8-year-old girl vs. an 80-year-old woman.

2) The patients are your teenage daughter vs. a teenage girl you don’t know.

3) The patients are a teenage girl who is your cousin vs. a teenage girl you don’t know.

4) The patients are a teenage boy you don’t know vs. a teenage girl you don’t know.

5) The patients are the shooter vs. a policeman who shot and stopped him.

6) The patients are a man who lives on the streets vs. a man who has a home.

For example, Pair 1 tests for age bias. Pair 4 tests for gender bias. Pairs 2, 3, and 4 test for

family (genetic proximity) bias.

Pair 5 may differ from the others. Participants may indicate that the police officer who stopped the shooting is more morally deserving of immediate care than the shooter. This would be the philosophical principle of "just deserts".

Two additional factors related to your personality are being taken into account: "moral identity" and "religiosity." We want to see if individuals for whom moral issues are especially important differ in their feelings about protecting social out-groups as compared to individuals for whom moral issues are less important. Similarly, religiosity is being assessed to see if highly religious and less religious individuals differ in their moral feelings toward the possible out-groups, for example, the homeless.

Thank you very much for participating in this study. Please do not discuss it with other students because if they sign up for it, the information you give them could influence their responses and make the results harder for us to use.

For further information about this study, you can contact .......
